# The Serpin Superfamily and Their Role in the Regulation and Dysfunction of Serine Protease Activity in COPD and Other Chronic Lung Diseases

**DOI:** 10.3390/ijms22126351

**Published:** 2021-06-14

**Authors:** Gillian A. Kelly-Robinson, James A. Reihill, Fionnuala T. Lundy, Lorcan P. McGarvey, John C. Lockhart, Gary J. Litherland, Keith D. Thornbury, S. Lorraine Martin

**Affiliations:** 1 School of Pharmacy, Queen’s University Belfast, Belfast, Northern Ireland BT9 7BL, UK; gkellyrobinson01@qub.ac.uk (G.A.K.-R.); j.reihill@qub.ac.uk (J.A.R.); 2 Wellcome-Wolfson Institute for Experimental Medicine, School of Medicine, Dentistry and Biomedical Sciences, Queen’s University Belfast, Belfast, Northern Ireland BT9 7BL, UK; f.lundy@qub.ac.uk (F.T.L.); l.mcgarvey@qub.ac.uk (L.P.M.); 3 Institute for Biomedical and Environmental Health Research, School of Health and Life Sciences, University of the West of Scotland, Paisley, Scotland PA1 2BE, UK; John.Lockhart@uws.ac.uk (J.C.L.); Gary.Litherland@uws.ac.uk (G.J.L.); 4 Smooth Muscle Research Centre, Dundalk Institute of Technology, A91 HRK2 Dundalk, Ireland; keith.thornbury@dkit.ie

**Keywords:** serine protease, serpin, protease inhibitor, chronic obstructive pulmonary disease (COPD), antiprotease

## Abstract

Chronic obstructive pulmonary disease (COPD) is a debilitating heterogeneous disease characterised by unregulated proteolytic destruction of lung tissue mediated via a protease-antiprotease imbalance. In COPD, the relationship between the neutrophil serine protease, neutrophil elastase, and its endogenous inhibitor, alpha-1-antitrypsin (AAT) is the best characterised. AAT belongs to a superfamily of serine protease inhibitors known as serpins. Advances in screening technologies have, however, resulted in many members of the serpin superfamily being identified as having differential expression across a multitude of chronic lung diseases compared to healthy individuals. Serpins exhibit a unique suicide-substrate mechanism of inhibition during which they undergo a dramatic conformational change to a more stable form. A limitation is that this also renders them susceptible to disease-causing mutations. Identification of the extent of their physiological/pathological role in the airways would allow further expansion of knowledge regarding the complexity of protease regulation in the lung and may provide wider opportunity for their use as therapeutics to aid the management of COPD and other chronic airways diseases.

## 1. Introduction

Chronic obstructive pulmonary disease (COPD) is a heterogeneous, progressive lung disease that is characterised by persistent respiratory problems, including chronic inflammation, mucociliary dysfunction and a reduction in airflow as a result of structural changes to the airways and destruction of the lung parenchyma. In 2019, COPD accounted for approximately 3.2 million deaths globally, equating to 6% of total global deaths [1]. COPD is ranked the 3rd global leading cause of death and one of the few that is continuing to increase in incidence [1,2]. This is in comparison to ischemic heart disease and stroke, that currently rank as the 1st and 2nd leading causes of death, respectively, which are projected to remain at a steady state [3]. COPD is predicted to continue to increase in coming years as a consequence of aging populations in high-income countries and an increased prevalence of smoking in developing countries. By 2060, it is forecast that COPD will account for 5.4 million deaths globally [2].

In health, proteases are key components of host defence and maintain tissue homeostasis within the lung. However, under pathological conditions such as COPD, the usually tightly regulated balance between proteases and antiproteases is disrupted leading to excessive and deleterious proteolysis. A typical characteristic of an acute infective exacerbation and a primary contributor to the protease-antiprotease imbalance in COPD airways is the influx of large numbers of neutrophils [4]. Neutrophil activation and degranulation cause the release of a high concentration of pre-activated neutrophil serine proteases (NSPs) to include neutrophil elastase (NE), proteinase 3 and cathepsin G, of which NE is the predominant activity. Levels of NE have also been shown to correlate with a decline in lung function [5].

Aberrant proteolysis is further favoured through the inactivation of endogenous protease inhibitors within the highly oxidative environment of a smoker’s lung [5,6]. Cigarette smoke contains a plethora of chemicals, including high concentrations of oxidising agents and reactive oxygen species (ROS) [7]. Alpha-1-antitrypsin (AAT), in particular is neutralised upon exposure to cigarette smoke due to the oxidation of a single critical methionine (Met^358^) residue within its reactive centre loop (RCL) [6,7,8]. A further confounder is that cigarette smoke promotes inflammation of the airways, which acts as a driving force to stimulate additional neutrophil recruitment. This causes a significant shift in the exquisite balance that is required to regulate the normal function of proteases. Dysregulation is therefore due to a combination of excessive protease levels and a loss of the protective benefits provided by inhibitor defences.

A protease-antiprotease imbalance is not unique to COPD; other chronic lung diseases such as cystic fibrosis (CF), asthma [9], idiopathic pulmonary fibrosis (IPF) [10], acute lung injury (ALI), non-CF bronchiectasis and acute respiratory distress syndrome (ARDS) [11,12,13] are also characterised by an imbalance favouring dysregulated protease activity which, in many cases, contributes to their pathogenesis and rate of disease progression.

## 2. Serine Proteases

In humans, proteases account for approximately 2% of the genome of which approximately one third are serine proteases [14,15,16]. As the most abundant class of proteases, with over 26,000 members spanning all biological kingdoms, serine proteases are further subdivided into 13 clans and 40 families with a large variation in the distribution of clans observed between species [17,18].

Serine proteases are a functionally diverse group of proteases with a broad range of activity and functions requiring strict physiological control. There are a number of mechanisms which control serine protease activity, including their expression as zymogens (pro-proteases). These zymogens require activation often as a part of a proteolytic cascade, the best characterised cascade being that of the coagulation system. NSP zymogens are, however, activated as the neutrophil matures in the bone marrow through the removal of an amino (*N*)-terminal dipeptide by the cysteine protease, dipeptidyl peptidase 1 (cathepsin C) which occurs during azurophilic granule assembly [19]. As such the NSPs are constitutively active upon arrival at the site of infection. Regulation must therefore come from environmental conditions, for example pH, or from the presence of a superfamily of endogenous proteins called serpins (serine protease inhibitors). Serpins act via a unique irreversible mechanism divergent from that of standard protease inhibitors [20]. They bind in a pseudo-substrate lock-and-key fashion and have a relatively broad specificity within subclasses of serine proteases. By forming a covalent bond with their target proteases a conformational change occurs in the active site resulting in effective enzyme inhibition and the “suicide” of the serpin [21].

## 3. The Serpin Superfamily

### 3.1. Classification

Serpins are the largest, most broadly distributed and functionally diverse superfamily of protease inhibitors. Similar to the serine proteases, they have been identified across all five kingdoms of life [22] and are sub-divided into 16 clades (A–P) based on their phylogenetic relationships [23]. To date, there have been no less than 37 serpins identified in humans, separated into nine clades (A–I) with multiple members within each clade. Clade A represents the largest group of human serpins, within which serpins are named SERPINXY; where X denotes the clade and Y the number within said clade. In most cases serpins also have alternative names by which they are more commonly known due to their discovery preceding this nomenclature. Serpins may also be classified as inhibitory or non-inhibitory based on their functionality and are involved in numerous physiological functions in many cellular compartments throughout the body.

Although originally thought to solely inhibit serine proteases, it has since been discovered that ‘cross-class’ inhibitors of proteases exist within the serpin family [24]. For example, a viral serpin, CrmA, can inhibit caspase-1 [25], and SERPINB3 can inhibit papain-like cysteine proteases cathepsins S, K and L [26]. The prokaryotic serpin, miropin, has also been identified as an inhibitor of a broad range of serine proteases, including neutrophil and pancreatic elastases, cathepsin G, plasmin, trypsin, subtilisin as well as the cysteine proteases cathepsin L and papain [27,28,29]. Additionally, a number of serpins can regulate biological functions without exhibiting any inhibitory activity, such as the regulation of hormone transport and blood pressure [30].

### 3.2. Structure and Function

**Figure 1:**
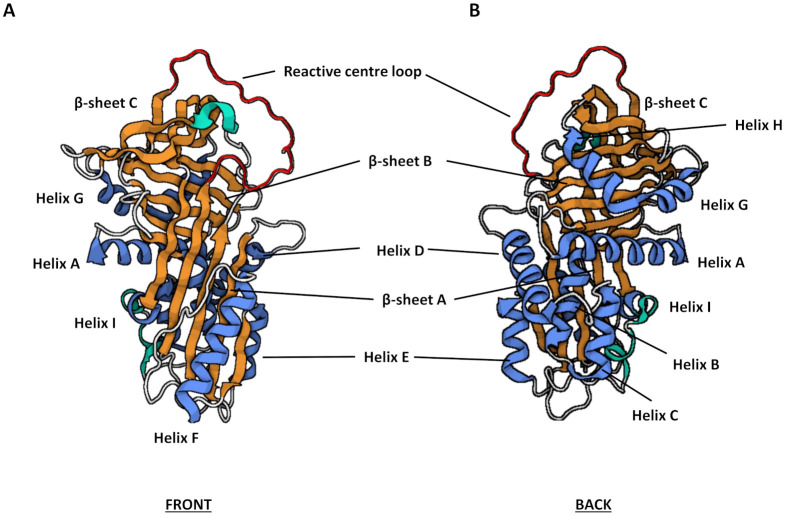
The canonical serpin tertiary structure (PDB code: 1QLP) comprises three β-sheets (orange), seven to nine α-helices (blue) and the reactive centre loop (red) shown in the front (**A**) and back (**B**) orientations. PDB code 1QLP was accessed on 25 March 2021. Created with BioRender.com.

Serpins are relatively large molecules, typically 350–400 amino acids in length and have an average molecular weight between 40–60 kDa [31], with variation in molecular weights a consequence of differential glycosylation. For example, SERPING1, also known as C1 inhibitor, has a particularly large molecular weight of 105 kDa due to a heavily O- and N-glycosylated extended N-terminus [32]. Highly conserved secondary and tertiary structures, however, contribute to a core domain consisting of three β-sheets (A–C), between seven and nine α-helices (A–I) and a RCL (Figure 1). The RCL forms an extended region that protrudes out from and above the body of the main serpin protein allowing for interaction with target proteases. This long flexible loop facilitates a conformational change during protease docking and inhibition, thus is critical in determining the specificity of serpin inhibition [30,33]. Indeed, the RCL along with the largest β-sheet, β-sheet A, comprise the key components of the core domain essential for serpin functionality.

Most serpins are extracellular (plasma) molecules and regulate proteases involved in blood coagulation, inflammation, tissue remodelling and the immune response. Serpins which reside primarily within cells tend to be expressed either ubiquitously or tissue-specifically [34]. These serpins usually belong to clade B and act on cytosolic processes and hence participate in cell-associated events [24].

### 3.3. Mechanism of Inhibition

Protease inhibition by serpins relies on the ability of the native proteins to exist in a metastable state that can readily transform to a more stable state during the inhibitory process. Central to the process is the RCL, which acts as a bait substrate to snare the target protease, which is then drawn into a conformational trap resulting in immediate inactivation. The initial interaction occurs when the target protease binds, as part of a reversible non-covalent Michaelis-Menten-like complex, to the P1 site, located on the exposed RCL of the serpin [35]. The docked protease then cleaves the scissile bond between the P1 and P1′ residues of the RCL, which results in a covalent bond being formed between the bound protease and serpin. At this point the reaction may complete the ‘inhibitory’ pathway, whereby the serpin and enzyme remain as a stable covalent (acyl-intermediate) complex, or an alternative ‘substrate’ pathway may be favoured. The inhibitory pathway progresses when the cleavage event results in a dramatic conformational change before the protease can complete what would normally be a product-forming deacylation step. The RCL-bound protease is pulled down into β-sheet A within the serpin core which facilitates an increase in thermal stability and releases the serpin from its metastable conformation [31]. The molecule thus transitions from a ‘stressed to relaxed’ state [33]. The formation of a covalent complex with the serpin consequently traps the target protease and both become inactivated.

The substrate pathway involves a quick dissociation of the protease from the Michaelis-Menten-like complex after cleavage of the RCL. This means that the protease escapes the conformational trap before loop insertion can take place. Furthermore, the liberated protease retains its activity whereas the cleaved, serpin is left inactive.

**Figure 2:**
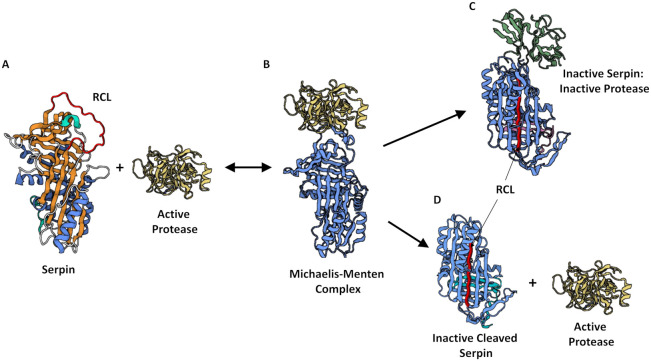
The inhibitory or alternative substrate mechanisms of serpin-protease interaction. (**A**) The metastable native serpin exists with its reactive centre loop (RCL) exposed as a pseudo-substrate for its target protease (PDB code: 1QLP). (**B**) The target protease binds to the P1 site located on the RCL forming a Michaelis-Menten complex (PDB code: 1OPH). (**C**) The target protease cleaves the target scissile bond inducing a dramatic conformational shift in the serpin resulting in the formation of a covalent serpin-protease complex where the RCL is pulled down into the centre of the serpin molecule and incorporated into β-sheet A, subsequently trapping and inactivating the covalently bound target protease (PDB code: 1EZX). (**D**) The “substrate” pathway will be favoured if the reaction does not occur rapidly enough. The protease escapes the conformational trap resulting in an inactive cleaved serpin and a free active protease (PDB code: 7API). PDB code 1QLP was accessed on 25 March 2021; 1OPH, 1EZX and 7API were accessed on 21 April 2021. Created with BioRender.com.

The requisite of RCL cleavage means that serpins are known as irreversible ‘suicide’ inhibitors [33]. The stoichiometry of inhibition (SI) is the ratio of moles of serpin required to inhibit 1 mole of protease [30] and is used to determine which pathway will predominate. SI should be close to 1 for effective inhibition to occur. The ‘suicide-substrate’ mechanisms by which serpins interact with a target protease are summarised in Figure 2.

### 3.4. Regulation of Function

There are a number of factors which enhance the inhibitory action of serpins including RCL length and flexibility [30], protease recognition and other exosite recognition sequences [31]. Some serpins also utilise co-factors to regulate activity, whereby the serpin still retains metastability and responsiveness, but the binding of the co-factor results in an optimal conformational change that confers maximal inhibitory activity where needed [34]. Common serpin cofactors are glycosaminoglycans (GAGs). GAGs are unbranched sulphated polysaccharide chains that are commonly utilised by some but not all serpins to modify inhibition kinetics. SERPINC1 (antithrombin III) shows increased inhibition of thrombin and factor Xa following activation by heparin, potentiating its anticoagulant effect 1000-fold [36]. Other well-known GAG-binding serpins include heparin co-factor II (SERPIND1) and protein C inhibitor (SERPINA5), which are capable of binding to heparin and heparan sulfate, with heparin co-factor II also able to bind dermatan sulfate [37]. For the most part, GAGs bind to serpins in a conserved region situated around helix D (Figure 1). SERPINA5 is the exception, with co-factor binding utilizing helix H [38].

### 3.5. Physiological Roles

In humans, 30 of the 37 serpins identified are inhibitory in function, of which many are critical to the tight control of biological processes that include the blood coagulation cascade, tissue remodelling, thrombosis and inflammatory responses [39]. Non-inhibitory functions have however, also been reported. These include hormone transport, as observed with cortisol binding globulin (CBG) (SERPINA6) [40] and thyroxine binding globulin (TBG) (SERPINA7) [41], molecular chaperoning (SERPINH7) [42] or tumour suppression (SERPINB5) [43].

### 3.6. Pathological Roles

The ability of serpins to undergo controlled conformational change to a more stable form is critical to their ability to carry out efficient inhibition. However, this feature can also render serpins susceptible to spontaneous rearrangements or mutations which can impair their affinity and effectiveness. Mutations can occur almost anywhere in the serpin which may convert the metastable native serpin to a more stable form exhibiting latency or inactive polymeric forms [44]. Some mutant serpins polymers are retained within the cell of synthesis and give rise to clinical conditions resulting from either protein overload (gain of function) leading to the death of the cell of synthesis, or loss of function through plasma deficiency of native serpins [45]. “Serpinopathies” is the term coined to describe pathologies manifesting from conformational discrepancies and polymerisation of serpins. There are numerous examples of serpinopathies. For example, emphysema and cirrhosis can be caused by deficiency and polymerisation of SERPINA1 [46], respectively, thrombosis may be caused by polymerisation of SERPINC1 [47], angioedema results from a deficiency of SERPING1 [48] and familial dementia is a consequence of SERPINI1 polymerisation [49].

## 4. Serpins Reported to Have Association with COPD or Other Chronic Airways Conditions

### 4.1. Clade A Serpins

Clade A serpins are extracellular, antitrypsin-like proteins that are encoded by genes on chromosome 14 [39]. Clade A is the largest clade of extracellular serpin and comprises 13 serpins which have both inhibitory and non-inhibitory functions, such as inflammatory response molecules (e.g., SERPINA1) and hormone-transport molecules (e.g., SERPINA7).

#### 4.1.1. SERPINA1

SERPINA1, also known as AAT, provides the main reservoir of protease inhibitory activity throughout the lung environment and is the archetypal member of the serpin superfamily. It is synthesised predominantly in the liver as a 52 kDa glycoprotein and is the most abundant protease inhibitor present in plasma (100 to 200 mg/dl) [50], although it has also been shown to be secreted from bronchial epithelial cells [51,52]. AAT is able to inhibit all of the NSPs but in particular, is a highly efficient inhibitor of NE with an association constant equal to 6.5 ± 4.0 × 10^7^ M^−1^ s^−1^ [53]. NE has a number of physiological substrates which include elastin and collagen, two components of the extracellular matrix, in addition to other proteins including defensins and immunoglobulin G (IgG). During periods of infection and inflammation, heightened NE activity also serves as a chemoattractant for the recruitment of other immune and inflammatory cell types, perpetuating protease release and proteolytic degradation of surrounding lung tissue. AAT is an acute phase protein whose role is to regulate the proteolytic activity of NE as well as cathepsin G and proteinase-3 to ensure that these proteases fulfil their roles pertaining to the inflammatory response, whilst preventing excessive connective tissue degradation [54].

AAT can also exert immune modulatory and anti-inflammatory functions. These activities are independent of its antiprotease activity and are based on specific molecular forms of AAT and associated binding partners, as demonstrated by Janciauskiene et al. [55]. AAT has been shown to lower levels of inflammatory signalling molecules released by macrophages as well as inducing changes in the expression of immune markers in pancreatic islet cells [56]. AAT can also be internalised by cells where it prevents cellular apoptosis via inhibition of caspase-3 [57]. It is also able to inhibit staurosporine (STS)-induced apoptosis thought to be mediated through a caspase-3 independent mechanism [58,59].

COPD that is associated with emphysematous changes in the lower airway is characterised by a protease-antiprotease imbalance of which the imbalance between NE and AAT is the best characterised. For some individuals a genetic deficiency in AAT can be identified as the driving force behind the development of early onset of COPD with risk substantially increased when circulating levels of AAT fall below the protective threshold of 11 µM (50 mg/dL). AAT deficiency was first described in 1963 by Laurell and Eriksson who noted a correlation between low serum levels of AAT and the incidence of emphysema [60].

AAT is encoded by the serine protease inhibitor clade A member 1 (*SERPINA1*) gene located on the long arm of chromosome 14 (14q32.13) [61] also referred to as the Protease inhibitor (Pi) locus. *SERPINA1* is 12.2 kilobases (kb) in size and contains seven exons, four coding and three non-coding and six introns [62]. Mutations in *SERPINA1* can prevent full translation of the protein (null mutations). Other mutations result in a translated protein that is misfolded, leading to varying degrees of deficiency [62,63].

AAT deficiency is an autosomal co-dominant trait, meaning that when one allele harbours the mutation, the phenotype is not as prominent as when both alleles are defective [64]. The most common alleles that are present in the general population are Pi*M, Pi*S and Pi*Z [62]. The nomenclature of the allele variants originates from their migration velocity through starch-gel electrophoresis; Pi*M migrated through the gel at a medium velocity, the Pi*S variant at slow velocity and the Pi*Z variant at a very slow velocity [62]. Pi*M is the wild type genotype, two copies of which contribute 100% of normal AAT serum levels [63]. The Pi*S mutant allele results from an A to T transversion at nucleotide position 9628, in exon 3. This mutation gives rise to a glutamic acid to valine amino acid substitution at amino acid 288 (E288V) [62], which contributes approximately 30% of functioning AAT in the circulation [51]. The mutant Pi*Z allele contributes approximately 10% of the normal protein function. The Pi*Z mutation is caused by a G to A transition at nucleotide position 11,940 in exon 5 and gives rise to an amino acid substitution from glutamic acid to lysine at amino acid 366 (E366K) [62]. The misfolded protein forms aggregates within the endoplasmic reticulum of hepatocytes, causing necrosis and ultimately cirrhosis of the liver tissue. The reduced levels of Pi*Z protein secreted into the blood stream also traffic to the lung where they continue to form pro-inflammatory polymers, further contributing to the heightened deficit of anti-elastase activity in the airways. Additionally, mutant AAT exhibits a reduced inhibition efficiency of NE with a rate of association of 2.7 ± 0.3 × 10^6^ M^−1^ s^−1^ [65,66]. Detrimental effects are further compounded by Pi*Z protein polymers acting as a chemoattractant for further neutrophil recruitment to the airways [67]. Individuals who harbour a mutant Z allele and a wild type (Pi*MZ) have about 60% of the circulating, functioning protein [51]. This is usually enough functioning protein to protect the lungs in non-smokers. If an individual carries two copies of the mutant alleles (Pi*ZZ) they will have between 15–20% of the functioning protein in circulation, which results in AAT deficiency [51]. Pi*ZZ individuals have a much higher risk of developing liver and lung diseases compared to Pi*MZ or Pi*MM individuals, however, some Pi*ZZ individuals may lead healthy lives without developing lung or liver disease [51].

In the native protein, methionine residues located at positions 351 and 358 can be readily oxidised resulting in inactivation of NE inhibitory activity [8,68,69]. It is postulated that oxidation of these methionine residues play a critical role in normal physiology. Oxidising agents released from neutrophils may ensure proximal AAT is deactivated to allow for the normal functioning of NE [8]. However, when external factors such as cigarette smoke, with its high concentration of oxidising agents, oxidise these methionine residues this can negatively affect the protease-antiprotease balance [70]. Moreover, smokers with AAT deficiency develop more severe COPD than non-smokers who also harbour the same AAT deficiency [61]. This is most likely a consequence of already limited levels of functionally active AAT being oxidised by cigarette smoke which neutralises any residual inhibitor protection. The result is increased NE-mediated emphysematous destruction.

#### 4.1.2. SERPINA3

SERPINA3, more commonly known as alpha-1-anti-chymotrypsin (ACT), is a heavily glycosylated protein with a molecular weight ranging between 55–66 kDa depending on the glycosylation status of the protein [71]. ACT inhibits many serine proteases such as chymotrypsin, mast cell chymases, kallikreins 2 and 3 and pancreatic cationic elastase [72,73,74,75,76]. SERPINA3 plays a role in immune and inflammatory responses through inhibition of chymotrypsin and cathepsin G [23]. ACT shows the greatest association with cathepsin G which is thought to be the main physiological target of the serpin [53]. ACT is an acute phase protein with normal circulating protein levels between 0.3 and 0.6 mg/mL which can surge fourfold in response to inflammation within eight hours of onset [77,78].

Two familial point mutations in the ACT gene have been associated with COPD [79]. The first mutation arises from a C→G transversion in exon III resulting in a proline residue being substituted for an alanine residue at position 229 (P229A) [80]. The second mutation arises from a T→C transition resulting in a substitution of a leucine residue to a proline residue at position 55 (L55P) [72]. The L55P mutation causes a conformational change to the protein resulting in a tertiary structure similar to the proposed inactive intermediate described between the latent and polymerised conformations in the mechanism of inhibition [81]. The resultant protein resembles the inactive serpin-protease complex depicted in Figure 2C, however, in this scenario the protease would be replaced by the RCL of another serpin. This conformational change promotes aggregation and retention in hepatocytes culminating in reduced circulating levels of the inhibitor, a similar observation to that of AAT deficiency. However, lung and liver disease manifestations are very rare in the general population [79]. This is because the observed ACT deficiency is only partial, as people heterozygous for ACT mutations retain inhibitor levels approximately 60% of normal protein levels and there have been no reports of homozygous individuals [82]. The rarity of the mutations in the general population have made it difficult to confirm any association with COPD [83] suggesting that while ACT may play a role in a small subset of individuals with COPD it is unlikely to be a significant contributor to the overall pathogenesis of COPD.

A characteristic unique to ACT, amongst the other human serpins and due to a series of lysine residues lying within close proximity to each other, is the ability to bind double stranded DNA independent to the inhibitory action of ACT [84]. The functional implication of this interaction remains to be determined [84].

#### 4.1.3. SERPINA5

SERPINA5, also known as protein C inhibitor (PCI), is a 57 kDa glycoprotein composed of 387 amino acids. PCI is found in a wide range of bodily fluids such as urine, tears, saliva, milk, amniotic fluid and seminal fluid [85]. PCI is a heparin-dependent serpin with broad protease specificity [86,87,88,89,90]. PCI exhibits both pro- and anti-coagulant activities dependent on the bound protease.

PCI shares the usual typical serpin structure, however, there are some noteworthy differences. The RCL of PCI is longer and exhibits more flexibility than other serpins which may account for the broad protease specificity [91]. Unlike the other heparin binding serpins, the heparin binding site of PCI is located along its highly basic helix H, as opposed to helix D [38].

The specific involvement of PCI in normal lung physiology is yet to be established but studies conducted to date suggest that PCI may play a protective role within the lung environment. Although expression of PCI in wild type mice is localised to the reproductive systems of both males and females, a transgenic mouse model overexpressing PCI developed by Hayashi et al. [92] has provided a useful tool to investigate the function of PCI in a variety of other tissues; using this transgenic model, Nishi et al. demonstrated that PCI protects against monocrotaline-induced hypertension in the lung [93]. It is thought that PCI elicits these protective effects via anti-inflammatory and anti-coagulant mechanisms [93,94]. While human studies centred around the role of PCI in the lung are lacking, elevated PCI levels have been identified in the bronchoalveolar lavage fluid (BALF) of patients with interstitial lung disease compared to healthy control subjects and were found to inversely correlate with fibrinolytic activity within the lung environment [95]. PCI can inhibit fibrinolysis directly or indirectly; direct inhibition is mediated via the suppression of plasminogen activator thus preventing the formation of plasmin. Whereas, indirect inhibition of fibrinolysis is modulated by blocking activated protein C (APC) activity [95].

#### 4.1.4. SERPINA7

SERPINA7, more commonly known as thyroxin-binding globulin (TBG), is a 54 kDa extracellular serpin with non-inhibitory function. TBG is one of three major thyroid hormone transport proteins along with transthyretin and serum albumin. TBG binds and transports triiodothyronine (T_3_) and thyroxine (T_4_) with a greater affinity than the other two transport proteins despite being the lowest abundant of the three [96].

To date, limited work has been carried out on the role of TBG in the lung environment; increased TBG levels have been associated with the severity of computed tomography-assessed emphysema [97]. Furthermore, TBG was identified as a novel plasma marker of COPD using isobaric tag for relative and absolute quantification (iTRAQ) proteomics [98]. Elevated plasma TBG levels were detected in patients with COPD compared to control subjects and correlated with reduced lung function and acute exacerbations [98].

#### 4.1.5. SERPINA8

SERPINA8, better known as angiotensinogen (AGT), is a non-inhibitory member of the clade A serpins. AGT can exist as a non-glycosylated protein with a molecular weight of 53 kDa or, dependent on how many of its four N-linked glycosylation sites are glycosylated, may have a molecular weight of up to 75 kDa [99]. AGT is the precursor of angiotensin peptides in the renin-angiotensin system making it a pivotal protein in blood pressure regulation.

A single nucleotide polymorphism (SNP) in the *SERPINA8* gene substitutes a methionine residue at position 235 for a threonine residue (M235T). Individuals homozygous for the M235T variant have increased circulating SERPINA8 levels, approximately 15% greater than individuals without the SNP. This SNP has been associated with conditions including hypertension, coronary heart disease and atrial fibrillation [100,101]. More recently, the SNP has been associated with higher plasma concentrations of AGT protein in COPD patients from a Turkish population relative to a control group [102].

#### 4.1.6. SERPINA12

SERPINA12, more commonly known as vaspin, is a 47 kDa protein composed of 415 amino acids. It is an extracellular, non-inhibitory serpin and insulin-sensitising adipocytokine [103]. Additionally, SERPINA12 exerts anti-inflammatory effects via inhibition of ROS and NF-κB (nuclear factor kappa B) signalling and activation of AMPK (5′ AMP-activated protein kinase) and Akt (previously known as Protein kinase B) signalling [104]. There have been limited reports of SERPINA12 in the lung environment, however, two independent studies have described an association of SERPINA12 with reduced lung function. Increased SERPINA12 was observed in a Korean population and correlated with low cardiorespiratory fitness, indicating it may have a role in the lung [105]. Despite the negative correlation of SERPINA12 with cardiorespiratory fitness, it is thought that SERPINA12 may have a protective role against lung injury potentially achieved through an anti-inflammatory effect [104]. Further to this, a rare SNP in the *SERPINA12* gene was associated with severity of airflow limitation in a COPD population [106], however, it is unreported as to what effect this SNP has on SERPINA12 expression.

### 4.2. Clade B Serpins

Clade B serpins consist of the intracellular, ov-serpins and are encoded by genes on chromosomes 6 and 18 [39]. Serpins B1, B6 and B9 play key roles in immune system function through inhibition of NE, cathepsin G and granzyme B [23]. This demonstrates that ov-serpins can target molecules which are also targeted by extracellular serpins, highlighting the dual role of the serpins; intracellular serpins having a cytoprotective effect while extracellular serpins protect the surrounding tissues [34]. Other inhibitory serpins within clade B include SERPINB3 and SERPINB4, which have been shown to increase mucus production by inducing transcription factors SPDEF (sterile alpha motif (SAM) pointed domain containing ETS transcription factor) and FOXA3 (forkhead box transcription factor A3) resulting in goblet cell hyperplasia [107].

#### 4.2.1. SERPINB1

SERPINB1, also known as leukocyte elastase inhibitor (LEI), is an intracellular inhibitory serpin. It can inhibit both the serine elastase-like and chymotrypsin-like proteases with inhibition against NE, cathepsin G, proteinase-3, chymase and chymotrypsin being reported [108]. This diverse protease specificity is possible via the presence of two different P1 sites present in the RCL. Elastase-like proteases target the classical P1 site, a cysteine residue at position 344, which corresponds to the P1 methionine residue at position 358 of AAT, whereas chymotrypsin-like proteases target the second P1 site, a phenylalanine residue at position 343 [108].

In mice, serpinb1 protected against *Pseudomonas aeruginosa* lung infection [109]. SERPINB1 is elevated in BALF of people with CF (PWCF) compared to normal control subjects. Moreover, levels of SERPINB1 were increased in BALF of PWCF who had active infection compared to uninfected PWCF [110]. SERPINB1 levels were also found to correlate with established biomarkers of inflammation in CF, neutrophil count, free NE and AAT bound NE (NE-AAT), indicating that SERPINB1 may be a suitable biomarker for inflammation in CF patients [110]. A role for SERPINB1 in COPD has not yet been reported.

#### 4.2.2. SERPINB2

SERPINB2, also known as plasminogen activator inhibitor-2 (PAI-2), exists mainly as a non-glycosylated intracellular serpin with a molecular weight of 47 kDa composed of 415 amino acids. However, it can be secreted as a heavier, 60 kDa, glycosylated protein [111]. It is a major intracellular inhibitor of tissue (tPA) and urokinase plasminogen activator (uPA).

Elevated PAI-2 levels were detected in alveolar macrophages of IPF patients compared with normal subjects, however, no difference in PAI-2 protein levels were observed in BAL supernatants of healthy and diseased cohorts [112]. *SERPINB2* gene expression is however, increased in bronchial brushings collected from asthmatics when compared with healthy controls and smokers (disease control) and is induced by IL-13 (interleukin-13) [113]. SERPINB2 along with chloride channel, calcium-activated, family member 1 (CLCA1) and periostin (POSTN), comprise a gene signature for type 2 high asthma [113,114,115].

#### 4.2.3. SERPINB3 and SERPINB4

SERPINB3 and SERPINB4, also known as squamous cell carcinoma antigen-1 and -2 (SCCA1/2), respectively, are evolutionary duplicated protease inhibitors sharing 98% of their nucleotide sequences and 92% of their amino acid sequences [116,117]. SERPINB3 and B4 are co-expressed in the lung [118]. SERPINB3 inhibits cysteine proteases cathepsin K, L, S and papain whereas SERPINB4 inhibits serine proteases cathepsin G and mast cell chymase [119]. SERPINB4 can inhibit cathepsin G but with a slower association rate constant (1 × 10^5^ M^−1^ s^−1^) than other serpins such as SERPINA1 (4 × 10^5^ M^−1^ s^−1^), SERPINA3 (5 × 10^7^ M^−1^ s^−1^), SERPINB1 (2.3 × 10^6^ M^−1^ s^−1^) and SERPINB6 (6.8 × 10^6^ M^−1^ s^−1^) [120,121].

Elevated levels of these inhibitors are detected during periods of inflammation which may indicate that they are upregulated to help suppress the inflammatory response. Equally their overexpression could indicate that they work to elicit a pro-inflammatory response, an effect supported by the observation of overexpression of these serpins in mice lacking the anti-inflammatory protein, uteroglobin [122]. The inflammatory Th-2 cytokines, IL-4 and IL-13, that are heavily involved in allergic asthma, can induce gene expression of SERPINB3 and SERPINB4 via STAT6 signalling [123]. Elevated SSCA levels are also found in the sera of patients with non-cancerous lung diseases such as asthma [123,124,125]. Smoking has also been reported to elevate SERPINB3 in the epithelial lining fluid of COPD patients [126].

#### 4.2.4. SERPINB6

SERPINB6, also known as proteinase inhibitor (PI)-6, is an intracellular serpin with inhibitory function. An arginine residue is present at the P1 site on the RCL of SERPINB6 which confers specificity for trypsin-like proteases including thrombin [127] and kallikrein-8 [128], however, its primary target is cathepsin G [121]. SERPINB6 is co-localised with cathepsin G in monocytes and granulocytes and is hypothesized to neutralize excessive cathepsin G activity within these cells following phagocytosis [121]. Additionally, abundant SERPINB6 expression is also observed in human mast cells where it is thought to interact with monomeric β-tryptase [129]. To date, literature describing a role for SERPINB6 within the airways is lacking. Interactions between SERPINB6 and proteases known to be involved in COPD (cathepsin G and β-tryptase), could infer an undescribed protease-antiprotease imbalance contributing to this disease.

#### 4.2.5. SERPINB9

SERPINB9 is more commonly known as PI-9 and is expressed within cytotoxic lymphocytes and dendritic cells [130,131]. SERPINB9 inhibits granzyme B [132], a serine protease, stored in granules within cytotoxic lymphocytes and natural killer cells [133], which activates apoptosis of target cells. SERPINB9 protects nearby cells from unintentional apoptosis by neutralizing granzyme B activity. Increased levels of cytotoxic lymphocytes are present in the lungs of COPD patient with cytotoxic potential increasing with disease severity [134,135]. Furthermore, elevated granzyme B levels have been detected in airways of current and ex-smoking COPD patients [136] which may facilitate disease progression via tissue damage. Currently, no relationship between SERPINB9 and COPD has been described, however, aberrant granzyme B activity suggests that there could be an imbalance at play which may contribute to pathogenesis.

#### 4.2.6. SERPINB10

SERPINB10, more commonly termed bomapin, is a 45 kDa protein comprised of 397 amino acids [137]. It is an inhibitory serpin capable of inhibiting trypsin and thrombin [137,138] and is localised to the nucleus of bronchial epithelial cells [139] due to the presence of a nuclear targeting domain [140]. Epithelial SERPINB10 mRNA and protein expression were found to be greatly increased in asthmatics compared to normal control subjects, correlating with both airway hyperresponsiveness and airway eosinophilia [139]. The role of SERPINB10 in allergic airway eosinophilic inflammation is thought to be elicited via modulation of periostin and CCL26 (chemokine (C-C motif) ligand 26) expression [139]. SERPINB10 expression is significantly associated with the aforementioned gene expression signature corresponding to type 2 high asthma [139]. 

Like SERPINB2, SERPINB10 expression can be stimulated by IL-13 [141].

### 4.3. Other Serpins

#### 4.3.1. SERPINE1

SERPINE1, more commonly referred to as plasminogen activator inhibitor-1 (PAI-1), is a 50 kDa glycoprotein composed of 379 amino acids. Under physiological conditions, PAI-1 is considered the main inhibitor of plasminogen activation due to its regulation of both tPA and uPA. PAI-1 can also inhibit APC [142] and thrombin [143] in the presence of cofactors heparin and vitronectin. PAI-1 regulates fibrinolysis through inhibition of the plasminogen activators. Fibrin deposition is characteristic of a number of lung diseases including IPF, ARDS and ALI. Investigations into the dysregulation of fibrinolysis have identified PAI-1 as a target of interest.

PAI-1 is expressed by multiple cell types in the lung environment and heightened levels of PAI-1 have been detected across numerous lung conditions. Increased PAI-1 levels were recorded in alveolar macrophages from patients with ARDS [144]. PAI-1 protein levels in BAL supernatant were significantly increased in IPF samples compared with normal control subjects [112]. BAL concentrations of PAI-1 were increased in patients with ventilator-associated pneumonia (VAP) [145,146].

Raised levels of PAI-1 are also present in asthmatics. A polymorphism (-675 4G/5G) in the promoter sequence of the PAI-1 gene, which causes a single guanosine deletion, is associated with a predisposition to developing asthma and is favourably transmitted to asthma patients [147,148]. The 4G polymorphism causes elevated PAI-1 protein levels which ultimately act to suppress fibrinolytic activity in the airways and accumulation of fibrin ensues. Increased circulating PAI-1 protein levels were associated with a decreased forced vital capacity (FVC) in asthma patients while no relationship with forced expiratory volume in 1 second (FEV_1_) was observed [149].

Smokers have increased PAI-1 levels compared to non-smokers [149]. Interestingly, nicotine was found to up-regulate PAI-1 in patients with COPD [150], with PAI-1 protein concentrations increased in sputum of COPD patients [151]. 

Blood protein levels of PAI-1 are increased in patients with idiopathic pulmonary arterial hypertension (PAH) [152] and heightened further in individuals with chronic thromboembolic PAH [153]. Hypoxia, a driver of PAH, induces overexpression of PAI-1 in smooth muscle cells of the lungs [153]. 

Increased PAI-1 serum concentrations were measured in SARS-CoV infected patients during the 2002/2003 epidemic [154]. Additionally, increased PAI-1 gene expression was observed in primates during infection with SARS-CoV [155]. SARS-CoV infection induces ALI which can develop into the more detrimental ARDS. More recently, the lungs of patients with COVID-19 (SARS-CoV-2 infection) exhibit fibrin depositions [156] indicating that PAI-1 may be a useful biomarker for risk of developing ARDS. 

Given that increased levels of PAI-1 are associated with numerous lung diseases it is not surprising that a deficiency in PAI-1 confers protection against excessive fibrin accumulation and bleomycin-induced fibrosis in the lungs [157,158,159]. Furthermore, PAI-1 is a target for therapies against lung injury resulting from fibrin deposition. In asthma, tiplaxtinin, a small molecule inhibitor of PAI-1 reduces airway remodelling in animal models of chronic asthma [160].

#### 4.3.2. SERPINE2

SERPINE2, also known as protease nexin I, is a 44 kDa glycoprotein. The main physiological role of SERPINE2 is to facilitate coagulation and fibrinolysis. SERPINE2 inhibits trypsin-like serine proteases, trypsin, thrombin, urokinase and plasmin but does not inhibit NE nor chymotrypsin-like proteases [161,162]. Extensive research has been conducted on SERPINE2 in the brain but there are no studies on the physiological role of SERPINE2 in the lung environment [163,164].

In contrast to the lack of information on a physiological role, multiple studies have described an association of SERPINE2 with lung pathophysiology. SERPINE2 gene expression was higher in people with severe emphysema compared to controls [165]. Whilst SNPs in SERPINE2 have also been associated with COPD, the role of SERPINE2 in this chronic lung disease has not been determined [165]. Multiple studies have investigated the association between SERPINE2 and the risk of COPD with inconsistent findings. DeMeo et al. discovered a significant association of 18 *SERPINE2* SNPs with spirometric phenotypes in a severe early-onset COPD cohort; five SNPs were associated with severe COPD from a Caucasian American population [165]. Interestingly, Chappell et al. found that five SNPs previously associated with severe COPD in the American population were not associated with a risk of COPD in a larger European population [166]. Alternatively, Zhu et al. associated five SERPINE2 SNPs with risk of COPD within a family-based study and a single SNP with a significantly increased risk of COPD in a case-control study from a Norwegian population [167]. A study conducted on a Korean population confirmed the association of this latter SNP with risk of COPD previously described in the Norwegian population, whereby this SNP was found to be associated with a significantly decreased risk of COPD [168]. Despite, the variation in reports surrounding the SERPINE2 gene it is evident that there is an as yet undefined role for the serpin in the pathogenesis of COPD. Further investigation would be required to elucidate the mechanism through which SERPINE2 acts.

#### 4.3.3. SERPINF1

SERPINF1, better known as pigment epithelium-derived factor (PEDF), is a 50 kDa glycoprotein belonging to the non-inhibitory serpins. PEDF is widely expressed throughout the human body and plays a role in numerous biological processes. It possesses tumour suppressive properties, angiostatic activity and neurotrophic capabilities. There are limited reports of the role of PEDF in the lung. However, increased expression has been identified in the lungs of patients with IPF [169], with aberrant angiogenesis implicated in disease pathogenesis [170]. Concentrated pockets of PEDF have been found in regions with reduced vascular density in IPF while observing reduced vascular endothelial growth factor (VEGF) concentrations [169]. This may be indicative of an imbalance between the pro-angiogenic activities of VEGF and the anti-angiogenic activities of PEDF.

## 5. Therapeutic Use of Serpins

Although it is evident that further investigations into the role of serpins in chronic airways disease is required, it is crucial to appreciate the potential these endogenous inhibitors hold. They can be therapeutically modulated to restore normal function and regulation of a number of biological pathways, or recombinant protein therapies can be developed to restore deficient proteins. The prime example of serpin replacement therapy is plasma-derived AAT augmentation for individuals with pulmonary disease associated with AAT deficiency [171].

AAT augmentation therapy studies have also been conducted in CF patients [172,173,174]. While CF patients do not possess the genetic mutation causing AAT deficiency, they do exhibit a protease-antiprotease imbalance favouring NE activity provoked by neutrophilic infiltration in response to cycles of chronic infection. Introduction of exogenous AAT suppresses excessive NE activity in CF lungs. Inhalation of recombinant AAT in CF patients is safe and well tolerated and allows for direct application of the inhibitor to the site of active proteolytic destruction while preventing systemic exposure and off-target effects [172,174]. Another example of serpin augmentation therapy is the use of recombinant human antithrombin replacement therapy in deficient individuals with clinical conditions that promote thrombosis [175].

Moreover, given their various roles in physiological and pathological processes, serpins have significant potential for therapeutic applications beyond replacement therapy. There are a number of mechanisms by which the serpin structure, function and dysfunction may be exploited for use, such as the inhibition or mimicking of serpin function as protease inhibitors, interference in polymerisation of pathological serpins or through exploitation of non-inhibitory functions. Thus, there is wide-ranging potential for future work in this area with engineered serpins holding promise in the treatment of a wide variety of diseases. An example to highlight this is the site-directed mutagenesis of AAT to develop an inhibitor of quite different specificity [176]. By increasing selectivity and potency of a serpin for a desired protease activity, the likelihood of off-target effects is reduced. However, to make use of serpins as therapeutic targets for treatment of chronic lung diseases further investigations into their (patho)physiological roles must be conducted.

## 6. Animal Models of Serpin-Associated Human Disease

From this review, it is evident that for the most part the (patho)physiological roles of the described serpins must be elucidated before their utilisation as therapeutic targets or exploitation as drug molecules. Animal models can be used to determine these roles across a broad range of diseases. Most commonly described serpin animal models are knockout mice. For example, *spi2A/serpinag3*, the mouse orthologue of *SERPINA3*, knockout mice have been used to study regulation of immune cells [177,178]. A *serpinc1* knockout mouse model allows the study of thrombosis-related coagulopathy [179]. A further example is the use of *serpinb1* knockout mice to investigate neutropenia [180]. Other animal models for the study of serpins do exist where *Serpinc1* knockout rats have facilitated investigations into acute kidney injury [181]. 

Although the use of animal models is crucial for the understanding of serpins in health and disease, species differences may complicate matters. For instance, wild type mice only express *serpina5* in their reproductive tracts [182], whereas humans ubiquitously express *SERPINA5*. Despite mice proving suitable models for investigating the role of SERPINA5 in the reproductive system, they are not relevant when studying other functions of the serpin. To overcome this problem a transgenic *serpina5* knock in mouse was established [92].

In addition to differences in serpin expression profiles, mice have 60 functional serpin genes compared to 37 in humans [23]. Many of these extra serpins are orthologues of human genes. Mice possess six *serpina1* paralogues which proved difficult when attempting to generate a *serpina1* knockout mouse model for the study of AAT deficiency. A mouse model of genetic emphysema was subsequently created in 2018 by knocking out five of the six paralogues via CRISPR/Cas9 [92].

## 7. Conclusions

Excessive serine protease activity is heavily implicated across numerous chronic lung diseases. It is, therefore, not surprising that serpins, the major endogenous regulators of serine proteases, are also associated with disease. A deficiency of the archetypical serpin, AAT, is well established as a contributing factor to emphysema and COPD pathogenesis albeit in small group of patients. Other protease-antiprotease imbalances are however, likely to be at play in other subgroups of COPD patients, identification of which will be a pre-requisite for the therapeutic correction of the previously undescribed imbalance. Further investigation into the role of the serpins identified to have an association with COPD may bridge this gap. Additionally, research into serpins that have been described as playing a role in other chronic airways diseases, such as SERPINA5 and SERPINB1, but conducted within a COPD setting, may also yield new knowledge. Interestingly, a number of serpins discussed in this review that correlate with disease are actually increased in expression which may be due to other factors over and above any protease inhibitory effect. It is largely evident that while serpins are dysregulated across chronic lung diseases, very little is known about how they functionally contribute to disease. Further investigations to uncover their full significance as regulators of protease activity in the lung or indeed any non-inhibitory effects are needed in order to assess clinical utility as either biomarkers of disease or therapeutic drug candidates.
